# Run4Love, a mHealth (WeChat-based) intervention to improve mental health of people living with HIV: a randomized controlled trial protocol

**DOI:** 10.1186/s12889-018-5693-1

**Published:** 2018-06-26

**Authors:** Yan Guo, Y. Alicia Hong, Jiaying Qiao, Zhimeng Xu, Hanxi Zhang, Chengbo Zeng, Weiping Cai, Linghua Li, Cong Liu, Yiran Li, Mengting Zhu, Nathan Asher Harris, Cui Yang

**Affiliations:** 1 School of Public Health, Sun Yat-sen University, #74 Zhongshan 2nd Road, Guangzhou, 510080 China; 2 Center for Migrant Health Policy, Sun Yat-sen University, #74 Zhongshan 2nd Road, Guangzhou, 510080 China; 3 Sun Yat-sen Global Health Institute, Institute of State Governance, Sun Yat-sen University, #74 Zhongshan 2nd Road, Guangzhou, 510080 China; 4 Department of Health Promotion and Community Health Sciences, School of Public Health, Texas A&M University, 212 Adriance Lab Road, College Station, TX 77843 USA; 5 Department of Infectious Disease, Guangzhou Number Eight People’s Hospital, #627 Dongfeng Road, Guangzhou, 510080 China; 6 Johns Hopkins Bloomberg School of Public Health Baltimore, 615 N. Wolfe Street, Baltimore, MD 21205 USA

**Keywords:** Mental health, mHealth intervention, People living with HIV (PLWH), Depression, Randomized controlled trial

## Abstract

**Background:**

People living with HIV (PLWH) suffer from high rates of mental illness; but targeted effective interventions are limited, especially in developing countries. High penetration of smartphone usage and widespread acceptance of social media applications provide an unprecedented opportunity for mobile-based health interventions (mHealth interventions) in resource-limited settings like China. The current report describes the design and sample characteristics of the Run4Love randomized controlled trial (RCT) aimed at improving mental health in PLWH in China.

**Methods:**

A total of 300 PLWH with elevated depressive symptoms were recruited and randomized into either the intervention or control group. Participants in the intervention group received an adapted cognitive-behavioral stress management (CBSM) course delivered by the enhanced WeChat platform (for 3 months) and were motivated to engage in physical activities. Progress of the participants was automatically tracked and monitored with timely feedback and rewards. The control group received a brochure on nutrition for PLWH in addition to standard care. The outcome assessments are conducted at baseline, 3, 6, and 9 months using tablets. The primary outcome is depressive symptoms measured by the scale of the Center for Epidemiology Studies Depression (CES-D). Secondary outcomes include quality of life, chronic stress measured with biomarker of hair cortisol, and other measures of stress and depression, self-efficacy, coping, HIV-related stigma, physical activity, and patient satisfaction. Mixed effects model with repeated measures (MMRM) will be used to analyze the intervention effects.

**Discussion:**

The Run4Love study is among the first efforts to develop and evaluate a multicomponent and integrated mHealth intervention to improve the mental health and quality of life of PLWH. Once proven effective, Run4Love could be scaled up and potentially integrated into the routine case management of PLWH and adapted to other populations with chronic diseases.

**Trial registration:**

Chinese Clinical Trial Registry - ChiCTR-IPR-17012606, registered on 07 September 2017.

## Background

People living with HIV (PLWH) are among those at high risk of mental illness, because of stigma associated with the disease and the groups with higher risks of infection and transmission of HIV [1]. Elevated mental health problems may lead to serious negative impacts on PLWH, including deteriorated immune system, worsened disease progression, and increased risky behaviors such as non-adherence to medication, inconsistent condom use, drug abuse, and suicide [2–7]. While life expectancy of PLWH has been vastly improved thanks to the development of anti-retroviral treatments, few effective interventions exist to improve mental health status of PLWH, especially in middle- and low-income countries, where more than 90% of PLWH live. Therefore, an urgent need exists for the development and evaluation of interventions to improve mental health status and quality of life for PLWH [8].

An estimated 758,000 PLWH live in China; similar to other middle- and low-income countries, the limited healthcare resources could not meet the increasing needs of PLWH for mental health services [9]. Only 20,000 certified mental health professionals practice in China, meaning 1.5 per 100,000 people, around 1/10 of the ratio in the US [10]. As a result of such limited resource of mental health service, only 8% of people with mental disorder ever sought professional help [11]. For PLWH, the stigma surrounding their conditions was much more severe and fewer have ever received any treatment or care for mental health issues. More than 95% of the Chinese adults own a mobile phone [12]. Such high coverage mobile access provides an unprecedented opportunity for mobile-based health (mHealth) interventions [13]. With over 900 million active users in 2017, WeChat is the most popular instant communication app and a de facto part of daily lives of most Chinese people [14].

Existing mHealth intervention studies in developed countries have shown feasibility and efficacy of improving mental health in different populations [15–19]. However, mHealth interventions for PLWH have mostly been focused on improving medication adherence, with limited data on their feasibility and efficacy to improve mental health of PLWH [20–22]. Moreover, existing mHealth studies were mostly pre-post designs without a control group or had small sample sizes; there is a lack of mHealth interventions with a rigorous design of randomized controlled trial (RCT) [17]. To address this literature gap, we developed the mHealth intervention “Run4Love” based on our pilot study and relevant literature. To the best of our knowledge, the Run4Love program is one of the first WeChat-based, multi-model mHealth interventions to improve mental health and quality of life of PLWH.

## Methods

### Overall study design

This intervention was designed for PLWH with elevated depressive symptoms. Eligible patients who provided informed consent were enrolled and, after a baseline interview and hair sample collection, randomized to the Run4Love intervention group or the waitlist control group. The intervention consists of information sending, monitoring, and timely feedback via the enhanced WeChat platform. Outcome assessments are conducted at baseline, 3, 6, and 9 months in the clinic. The 3-month assessment interval directly parallels the interval period between clinic visits by the participants. The primary outcome is depressive symptoms measured by the Center for Epidemiological Studies Depression Scale (CES-D). Secondary outcomes include quality of life, chronic stress (measured by hair cortisol), other depression and stress measurements, self-efficacy, coping, HIV-related stigma, physical activity, and patient satisfaction. The intervention protocol was approved by the Institutional Review Board at the Sun Yat-Sen University, and the study protocol has been registered in chictr.org.cn (number ChiCTR-IPR-17012606).

### Participation eligibility

The participation eligibility criteria were: 1) 18 years or older, 2) HIV seropositive, 3) having elevated depressive symptoms (CES-D ≥ 16), 4) willing to provide hair samples, and 5) using WeChat. Individuals were excluded if they: 1) were using psychiatric drugs, 2) had hair permed or dyed in the past 3 months, 3) were unable to finish the questionnaire due to mental or other illnesses or other reasons; 4) were unable to read or listen to the materials sent via WeChat (i.e., short articles, audios, posters); or 5) were unable to engage in physical activities due to medical reasons.

### Recruitment and randomization

Participants were recruited from the outpatient clinic of the only hospital designated for HIV treatment in Guangzhou, the third largest city in China. Patients waiting to see a physician were invited by the research staff to complete a brief screening questionnaire. Those who met the eligibility criteria were given a pamphlet with description of the Run4Love study and invited to join the study. Patients with interest to participate would receive further information about the study in a private room. After providing the written informed consent, eligible patients completed a baseline survey on a tablet and provided their hair samples.

Allocation to treatment group was carried out by a computer-generated randomization list with block size of 4 using SAS software version 9.4 (SAS Institute, Inc., Cary, NC, USA). Study participants were reimbursed 50 RMB (about 8 US dollars) or gifts of equivalent value for each of the study interviews completed (baseline and 3 follow-up interviews) and continued to receive all their standard HIV medical care.

### Intervention protocol

Participants in the intervention group received a 3-month mHealth intervention of the Run4Love, consisting of the following two major components: the adapted cognitive-behavioral stress management (CBSM) course and regular physical activity promotions delivered by our enhanced WeChat platform. We created a public account of Run4Love to deliver CBSM information; additional functions of automatic tracking of information reception and physical activity and instant feedback were added. Only participants in the intervention group could receive information from the Run4Love account. The four major components of the Run4Love protocol are detailed below.

#### Cognitive-behavioral stress management course

The cognitive-behavioral stress management (CBSM) course, an evidence-based intervention to improve mental health of PLWH was adapted for the Chinese PLWH on the social media platform of WeChat [23, 24]. Our bilingual research staff translated the content of CBSM; extensive formative research was conducted to ensure its cultural relevance. The program was piloted and further refined before its delivery. The final program consists of 9 sessions and 3 review sessions for a total length of 12 weeks (3 months); see Table 1 for details of adaption and program outlines.

**Table 1:** The adapted 9-session Cognitive-Behavioral Stress-Management (CBSM) course

Session	Content	Week
Session 1	Introduction to the program and stressors and stress responses	1
	Audio Progressive muscle relaxation for 16 muscle groups	
Session 2	Stress and awareness	2
	Audio Progressive muscle relaxation for 8 muscle groups	
Session 3	Negative thinking and cognitive distortions	3
	Audio Breathing, imagery, passive progressive muscle relaxation for 4 muscle groups	
Review 1	Review and course evaluation of session 1, 2, and 3	4
Session 4	Anxiety and depression	5
	Audio Breathing, imagery, passive progressive muscle relaxation	
Session 5	Rational thought replacement	6
	Audio Autogenic training for heaviness and warmth	
Session 6	Productive coping and executing effective coping responses	7
	Audio Autogenic training for heartbeat, breathing, abdomen, and forehead	
Review 2	Review and course evaluation of session 4, 5, and 6	8
Session 7	Introduction of meditation	9
	Audio Autogenic training with imagery and self-suggestions	
Session 8	Anger management	10
	Audio Mantra meditation	
Session 9	Social support and review of the program	11
	Audio Imagery and meditation	
Review 3	Review and course evaluation of all sessions	12

#### Physical activity promotion

The second major component of the Run4Love program was the promotion of regular physical activity. Information about the benefits of regular exercise, how to exercise safely and effectively, and healthy dietary information was sent via the Run4Love account on a weekly basis. One week after the recruitment, we called the participants to confirm their enrollment and assisted them to set goals for weekly physical activities (e.g., walk 8000 steps a day). They also learned to adjust their exercise routine based on personalized feedback sent via WeChat.

#### Automatic progress monitoring

**Fig. 1:**
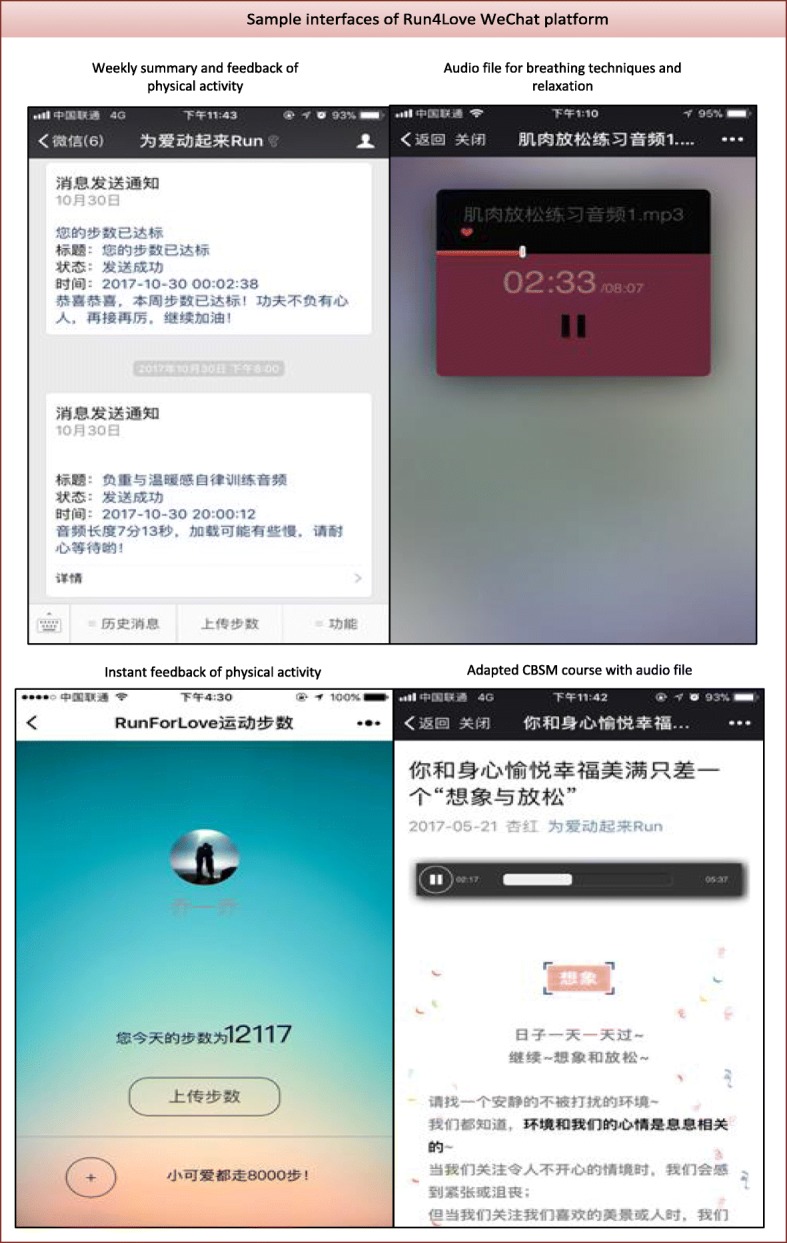
WeChat users’ interfaces in the Run4Love study

Participants receive CBSM and physical activity promotion information (including audio, pictures, and short essays) on their WeChat account 3–4 times a week based on the time slots they selected. The Run4Love platform could trace the frequency of reading or listening to each file. Based on frequency analysis, the most popular files would be repeatedly sent in the next 3 months post-intervention. Participants’ daily steps are also automatically tracked by the WeChat platform so we could send them instant and personalized feedback. Each week, based on their progress, we sent their weekly feedback with small incentives (e.g., red packet with small amount of money) via WeChat, see Fig. 1 for a sample interface of Run4Love platform.

#### Social support

Participants in the intervention group also received up to 5 phone calls from the research staff at one week, 1, 2, 5, and 8 months after enrollment. The phone call in the first week was for confirming participation and ensuring participants’ proper use of the system. The purposes of the phone calls are: 1) to provide feedback on participants’ mental health status based on their monthly quick assessment on WeChat; 2) to evaluate participants’ progress and help identify barriers, as well as to aid in finishing CBSM and engaging in regular physical activities; 3) to prevent and identify adverse events during the intervention and take appropriate actions; and 4) to provide reminders of their regular medical check-ups.

### Control group

Participants in the waitlist control group received a brochure on nutrition and healthy living in addition to standard care for HIV treatment. Our pilot study found that PLWH were quite concerned with their physical health conditions, and that nutrition information was well received by them. Participants in the control group would receive the Run4Love intervention after the completion of the trial.

### Outcome measures and data collection procedure

Table 2 summarizes the outcome measures, measurement variables/scales, internal reliability (Cronbach’s coefficient alpha) of the scales, and assessment times of the current study. Participants’ demographics, psychosocial outcomes, and satisfaction with the program would be collected with surveys on tablets. The primary outcome is depressive symptoms assessed at 3-month follow-up; it is measured by the Center for Epidemiological Studies Depression Scale (CES-D), Chinese version [25]. CES-D assesses participants’ mood in the past week, with 20 items measuring 4 dimensions (i.e., positive affect, depressed affect, interpersonal relationship, and somatic and retarded activity). The Cronbach alpha of the scale is 0.9. The scores of CES-D range from 0 to 60, with scores≥16 being considered as having elevated depressive symptoms and higher scores indicating more severe depressive symptoms.

**Table 2:** Measures and schedule of Run4Love outcome assessments

				Schedule					
Domain	Measure	Items	Alpha^a^	0 mo	1 mo	2 mo	3 mo	6 mo	9 mo
Demographics	Age, gender, height, weight, education, sexual orientation, marital status, employment, daily expenses affordability, HIV infection duration	10	n/a	X					
Depression	Center for Epidemiological Studies Depression Scale (CES-D)^b^	20	0.90	X			X	X	X
	Patient Health Questionnaire (PHQ-9)^c^	9	0.86	X	X	X	X	X	X
Physical activity	Global Physical Activity Questionnaire (GPAQ)^d^	16	0.85	X			X	X	X
Quality of Life	World Health Organization Quality of Life HIV short version (WHOQOL-HIV BREF)^e^	31	0.85	X			X	X	X
Self-efficacy	General Self-efficacy Scale (GSES)^f^	10	0.87	X			X	X	X
Stress	Perceived Stress Scale (PSS)^g^	10	0.83	X			X	X	X
	Hair cortisol (biomarker)	n/a	n/a	X			X		
HIV-related stigma	HIV Stigma Scale^h^	14		X			X	X	X
	*Internalized stigma (8 items)		0.92						
	*Perceived stigma (6 items)		0.90						
Coping	Simplified Ways of Coping Questionnaire (SWCQ)^i^	20		X			X	X	X
	*Positive coping (12 items)		0.89						
	*Negative coping (6 items)		0.78						
Satisfaction	Intervention-specific satisfaction	20	n/a				X		

^a^Cronbach’s coefficient alpha for internal reliability of the scale; n/a = not applicable

^b^CES-D scores range 0–27; higher scores indicate more severe depressive symptoms

^c^PHQ-9 scores range 0–60; higher scores indicate more severe depressive symptoms

^d^GPAQ uses Metabolic equivalents (METs) to measure the intensity of physical activities; higher METs indicate more intensive physical activities

^e^WHOQOL-HIV BREF scores range 24–120; higher scores indicate better quality of life

^f^GSES scores range 0–40; higher scores indicate better self-efficacy

^g^PSS scores range 0–40; higher scores indicate more stress

^h^HIV Stigma Scale scores range 14–56; higher scores indicate more HIV-related stigma

^i^SWCQ scores range 0–36 for positive coping and 0–24 for negative coping; higher scores indicate higher levels of coping

Secondary outcomes include quality of life, chronic stress (measured by hair cortisol), physical activity, other depression and stress measurements, self-efficacy, coping, HIV-related stigma, and patient satisfaction.

Quality of life is assessed by World Health Organization Quality of Life HIV short version (WHOQOL-HIV BREF), with 31 items measuring 6 domains (i.e., physical, psychological, level of independence, social relationships, environment, and beliefs) [26]. The scores of WHOQOL-HIV BREF range from 24 to 120 with a higher score representing a higher level of quality of life.

As a biomarker of chronic stress, hair samples are collected by research staff at baseline and the immediate follow-up after 3-month intervention. Following the procedure detailed by Veldhorst and colleagues, approximately 100 pieces of hairs from each participant are cut from the posterior vertex of the scalp, wrapped by tin foil, and stored at − 20 degrees Celsius temperature in fridge [27]. A commercially available enzyme-linked immunosorbent assay kit for salivary cortisol will be used to assess hair cortisol concentration for chronic stress. Higher levels of hair cortisol concentration indicate higher levels of chronic stress [28–30].

Physical activity is measured by the Chinese version of Global Physical Activity Questionnaire (GPAQ) that is widely used in people with chronic diseases [31]. Metabolic equivalents (METs) calculated from GPAQ measure the intensity of physical activities with METs≥600 representing that individuals meet the minimum requirement of WHO recommendation of weekly exercises intensity.

We also collected other psychosocial outcomes including the depression scale of Patient Health Questionnaire (PHQ-9) and Perceived Stress Scale (PSS) [32, 33]. The cut-off point for depression is 10 in PHQ-9 with higher scores indicating higher levels of depression. Self-efficacy is measured by 10-item General Self-efficacy Scale (GSES), Chinese version [34]. The total scores of GSES range 10–40 with higher scores indicating higher self-efficacy. Coping is assessed with Simplified Ways of Coping Questionnaire (SWCQ), Chinese version, with 20 items (scores 0–60) measuring 2 domains (i.e., positive and negative coping) [35]. HIV-related stigma is assessed by 14 items derived from the HIV Stigma Scale measuring internalized and perceived stigma [36, 37] with higher scores representing higher levels of stigma. Patient satisfaction will also be assessed with 20 items measuring general and intervention specific satisfaction, which will be collected in the follow-up surveys [38].

### Statistical considerations

#### Sample size justification

The sample size was calculated based on the primary outcome of depressive symptoms measured by CES-D. Effect size is widely used to measure treatment effects of continuous outcomes, calculated as the difference between groups divided by the pooled standard deviation [39]. A prior study of online intervention to treat depression using CES-D as the primary outcome reported the effect size of 0.57 [40]. Effect sizes of 0.2 and 0.5 are typically considered as the cut-off points for small and moderate effects [39]. To ensure enough sample size to detect moderate treatment effect, we hypothesized a conservative effect size of 0.4. Assuming *α* = 0.05, *β* = 0.15, and allowing for up to 20% attrition, the sample size of 282 is needed for the current study. Thus the 300 participants we recruited for this RCT were sufficient to detect the hypothesized effect size.

#### Statistical analysis

Baseline sample characteristics will be compared between the intervention and control groups using analyses of variance (ANOVA) for normally-distributed continuous variables, Wilcoxon tests for non-normally-distributed continuous variables, and Chi-square tests for categorical variables. Analyses of outcomes are based on intention-to-treat strategy for all randomized participants. For the primary outcome, group differences in CES-D scores over the 9 months of the trial will be estimated using mixed effects model repeated measures (MMRM) analysis, adjusting for baseline CES-D score and time [41]. As we collect data using a tablet during each interview, every question in the electronic questionnaire is required, so missing data is minimized. For respondents missing one or more interviews, MMRM allows data from completed interviews to be retained for analyses. For sensitivity analyses, group differences will also be compared using last-observation carried forward (LOCF) imputation for all outcomes [42].

A similar approach will be conducted to examine group differences in the secondary outcomes such as HIV-related quality of life and chronic stress shown in Table 2. Analyses on secondary outcomes will not be adjusted for multiple comparisons. Instead, results from secondary outcomes will be interpreted with caution unless they reach a high level of statistical significance (*P* < 0.001). Analyses will be performed using SAS Version 9.4 (SAS Institute, Cary, North Carolina).

### Baseline characteristics

**Fig. 2:**
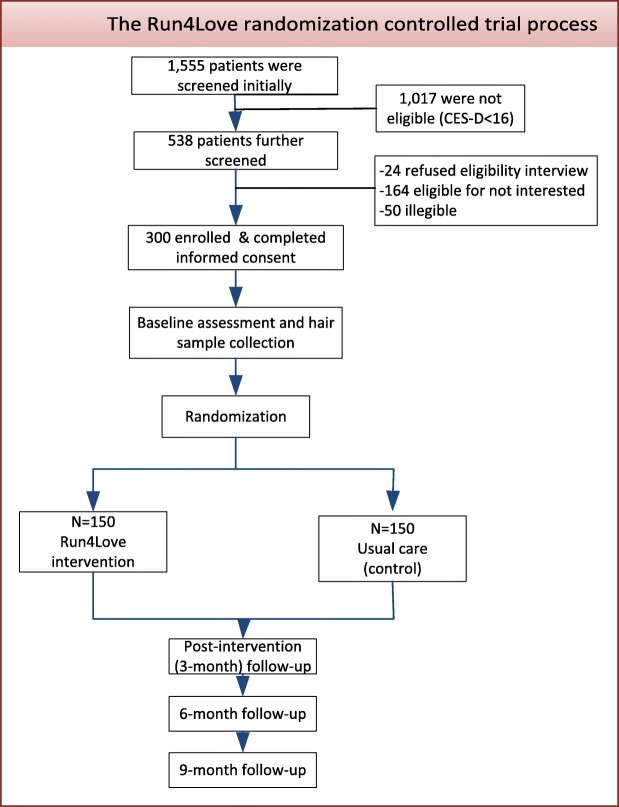
Flowchart of Run4Love intervention trial

Figure 2 shows the process of participant screening, eligibility determination, and enrollment conducted from September to December 2017. A total of 1555 PLWH were screened, with 538 having elevated depressive symptoms (CES-D ≥ 16). Twenty-four of the 538 patients were not interested in eligibility interview; 164 were eligible but not interested in the trial; and 50 were not eligible after further screening. Finally, 300 eligible patients consented and were enrolled; they were randomized to the intervention or control group. Randomization resulted in comparable groups on all variables except for sexual orientation (Table 3). Overall, the sample had a median age of 27.5 years (IQR: 24.5–51.3) and a median body mass index (BMI) of 20.1; the majority (92.3%) were men, well-educated (60.7% with at least some college education), unmarried (87.3%), employed (83.7%), with adequate income (85%), and most (81.7%) had sexual orientation of homosexual, bisexual, or uncertain. The participants had a median duration of HIV infection since diagnosis of 1.72 years (IQR: 0.59–3.71).

**Table 3:** Baseline characteristics of 300 participants enrolled in the Run4Love study

	Total (*n* = 300)	Intervention (*n* = 150)	Control (*n* = 150)
Age in years, median (Q1,Q3)	27.5 (24.5, 31.3)	27.4 (24.3, 31.1)	27.8 (24.6, 32.2)
Sex, % men	92.3	94.7	90.0
BMI, median (Q1,Q3)	20.1 (18.6, 21.7)	20.2 (18.7, 21.9)	19.9 (18.6, 21.3)
Education, % > high school	60.7	65.3	65.0
Homosexual/bisexual/uncertain,%	81.7	86.7	76.7
Married, %	12.7	12.0	13.3
Employed, %	83.7	82.0	85.3
Daily expenses, % affordable	85.0	86.0	84.0
Infection period in years, Median (Q1,Q3)	1.7 (0.6, 3.7)	1.7 (0.6,4.0)	1.8 (0.6,3.6)
CES-D [0–60], Median (Q1,Q3)	23.0 (19.0, 28.0)	23 (19.0, 28.0)	23 (19.0, 27.0)
PHQ-9 depression [0–27] Median (Q1,Q3)	10.0 (7.0, 13.0)	10.0 (7.0, 12.8)	10.0 (7.0, 13.0)
GPAQ physical activity (METs), % ≥600	43.3	43.3	43.3
HIV-related quality of life [24–120], Mean ± Standard deviation	77.0 ± 9.2	77.4 ± 9.0	76.6 ± 9.4
GSES Self efficacy [10–40], Median (Q1,Q3)	23.0 (20.0, 27.2)	23.0 (20.0, 28.0)	23.0 (20.0, 27.0)
Perceived stress [0–40], Median (Q1,Q3)	20 (18, 23)	20 (17, 22)	20 (18, 23)
HIV-related Stigma [14–56], Median (Q1,Q3)	38.0 (32.0, 42.0)	37.0 (32.0, 42.0)	38.0 (34.0, 42.0)
Positive coping [0–36], Median (Q1,Q3)	18 (14, 22)	18 (14, 21)	18 (13.2, 22)
Negative coping [0–24], Median (Q1,Q3)	12 (9, 15)	12 (9, 15)	12 (9, 15)
CD4, median (Q1,Q3)	413 (304, 534)	416 (309, 536)	405 (303, 522)

At baseline, the median score of CES-D was 23 (IQR: 19–28), representing moderate to severe level of depression (cut-off point for severe depression being 24). The median score of PHQ-9 was 10.0 (IQR: 7.0–13.0), meaning half of the participants reached clinical significance of depression. Perceived stress was 20 (IQR: 18–23), with the majority (85.3%) of the participants reporting moderate stress, 8.5% severe stress, and 6.3% mild stress. Less than half (43.3%) of the participants had met the WHO recommendations on physical activities with METs≥600. There was no significant difference between the intervention and control group in terms of psychological outcomes and physical activities (Table 3).

## Discussion

The Run4Love study achieved the targeted enrollment of 300 PLWH with elevated depressive symptoms. Most participants were willing to participate in the study. There was no significant difference in the CES-D scores between PLWH who were eligible but not interested and those enrolled in the study. Randomization resulted in comparable intervention and control groups in all measures except sexual orientation. Important innovations of the Run4Love study include the development of an enhanced WeChat platform, the multi-model intervention design, and testing hair cortisol as a biomarker of chronic stress. The enhanced WeChat platform was developed with the functions to automatically distribute multi-media intervention materials, track participants’ progress with those materials, and provide timely feedback. The Run4Love study has two major components: the CBSM program and physical activity promotions, coupled with timely feedback provided both automatically by the enhanced platform and by research staff, with both messages of encouragement and financial incentives. The Run4Love study is adaptable and suitable for resource-limited settings with constrained mental health services and has the potential to be scaled up. As the number of people living with chronic conditions has increased in recent years and chronic diseases account for more than 80% of deaths in China, the experience learned from the Run4Love study will also be valuable to manage other chronic conditions and reduce healthcare cost in society [43].

There are several limitations in the Run4Love study. First, all participants were recruited from one hospital in a large city, thus the sample might not be representative of the PLWH population in China, especially those from rural areas, or whose HIV seropositive status was unknown, or those not on HIV treatment. However, the recruitment site is the only hospital designated for HIV treatment in Guangzhou metropolitan area, serving more than 100,000 HIV/AIDS patients in the region. Second, the participants consist of mostly young men who have sex with men (MSM), which may be due to the fact that young MSM are better educated and more likely to accept a mHealth intervention. Further, existing studies have revealed that HIV seropositive MSM are more likely to experience depression and have a dire need for targeted mental health intervention [44]. Third, a potential self-report bias exists as most outcomes are measured using self-report scales. However, our RCT design could limit such bias, plus the biomarker of chronic stress measured by hair cortisol could provide more objective measurement.

The Run4Love study is among the first efforts to innovatively develop and test a social media-based mHealth intervention to improve psychological well-being and quality of life in PLWH. Once proven effective, Run4Love could be potentially integrated into the routine case management of PLWH. An intervention like Run4Love could also be adapted and tailored to other populations with chronic diseases where depression is prevalent (e.g., cancer survivors, postpartum women with depression). The follow-ups are currently ongoing and we will report the intervention effects when the follow-ups conclude.

## Abbreviations

AIDS: Acquired Immune Deficiency Syndrome
ANOVA: Analyses of Variance
BMI: Body Mass Index
CBSM: Cognitive-Behavioral Stress Management
CES-D: Center for Epidemiology Studies Depression
GPAQ: Global Physical Activity Questionnaire
GSES: General Self-Efficacy Scale
HIV: Human Immunodeficiency Virus
IQR: Interquartile range
LOCF: Last-Observation Carried Forward
MET: Metabolic equivalents
mHealth: Mobile Health
MMRM: Mixed Effects Model with Repeated Measures
MSM: Men Who Have Sex with Men
PHQ-9: Patient Health Questionnaire
PLWH: People Living with HIV
PSS: Perceived Stress Scale
RCT: Randomized Controlled Trial
SWCQ: Simplified Ways of Coping Questionnaire
WHO: World Health Organization
WHOQOL-HIV BREF: World Health Organization Quality of Life HIV short version
